# The Temporal Dynamics of Scene Processing: A Multifaceted EEG Investigation

**DOI:** 10.1523/ENEURO.0139-16.2016

**Published:** 2016-09-27

**Authors:** Assaf Harel, Iris I. A. Groen, Dwight J. Kravitz, Leon Y. Deouell, Chris I. Baker

**Affiliations:** 1 Section on Learning and Plasticity, Laboratory of Brain and Cognition, National Institute of Mental Health/National Institutes of Health, Bethesda, Maryland 20892; 2 Department of Psychology, The George Washington University, Washington, DC 20052; 3 Department of Psychology, The Hebrew University of Jerusalem, Jerusalem 91905, Israel; 4 Edmond and Lily Safra Center for Brain Sciences, The Hebrew University of Jerusalem, Jerusalem 9190401, Israel

**Keywords:** EEG, ERP, scene recognition, visual perception

## Abstract

Our remarkable ability to process complex visual scenes is supported by a network of scene-selective cortical regions. Despite growing knowledge about the scene representation in these regions, much less is known about the temporal dynamics with which these representations emerge. We conducted two experiments aimed at identifying and characterizing the earliest markers of scene-specific processing. In the first experiment, human participants viewed images of scenes, faces, and everyday objects while event-related potentials (ERPs) were recorded. We found that the first ERP component to evince a significantly stronger response to scenes than the other categories was the P2, peaking ∼220 ms after stimulus onset. To establish that the P2 component reflects scene-specific processing, in the second experiment, we recorded ERPs while the participants viewed diverse real-world scenes spanning the following three global scene properties: spatial expanse (open/closed), relative distance (near/far), and naturalness (man-made/natural). We found that P2 amplitude was sensitive to these scene properties at both the categorical level, distinguishing between open and closed natural scenes, as well as at the single-image level, reflecting both computationally derived scene statistics and behavioral ratings of naturalness and spatial expanse. Together, these results establish the P2 as an ERP marker for scene processing, and demonstrate that scene-specific global information is available in the neural response as early as 220 ms.

## Significance Statement

Humans can process complex scenes very rapidly and efficiently. While recent years have shown great progress in understanding where in the brain scene processing occurs, it is still unknown when in the brain scene-specific processing occurs. We describe a novel electrophysiological signature of scene-selective processing, the P2 event-related potential component. We found that P2, which peaks at ∼220 ms after stimulus onset shows a greater response to scenes than other categories, and distinguishes between scene images based on their global diagnostic properties, such as naturalness and spatial layout. Our findings, therefore, provide critical insight about the time course of scene processing, as they demonstrate that diagnostic scene information can be found as early as 220 ms after stimulus onset.

## Introduction

Real-world visual scenes are rich and complex, containing many different sources of information including spatial layout, local objects, and semantic associations. Despite this complexity, humans recognize scenes easily and very rapidly (Potter, 1976; Joubert et al., 2007; Greene and Oliva, 2009b). Functional magnetic resonance imaging (fMRI) studies have revealed a network of cortical regions engaged by scene processing that exhibit selectively higher responses to scenes than to other categories, such as faces and objects (Epstein, 2008). This cortical specialization likely reflects the unique physical properties and information contained in scenes relative to other visual categories. For example, visual scenes depict heterogeneous real-world environments, which often contain large-scale elements, such as walls, mountains, and buildings, that determine both the spatial layout and scene category (Henderson and Hollingworth, 1999). Consequently, scenes are often recognized based on their global distribution of information (Oliva and Torralba, 2001; Greene and Oliva, 2009a). In comparison, faces are very homogenous, sharing a small set of features organized in a prototypical configuration (Bruce et al., 1998).

Despite a growing understanding from fMRI of the regions that contribute to scene processing and the representations contained therein, its temporal dynamics remain unclear. To date, only a few studies have used electrophysiological measures to study the time course of global scene processing (Sato et al., 1999; Rivolta et al., 2012; Bastin, et al., 2013a; Groen et al., 2013, 2016a; Cichy et al., 2016). The scarcity of electrophysiological studies of scene processing is surprising given the large number of studies in the event-related potential (ERP) literature on face processing, and particularly on the face-selective N170 component (for review, see Rossion and Jacques, 2011). Two previous magnetoencephalography (MEG) studies attempting to find a “scene analog” to the N170 by contrasting responses to faces and scenes have reported inconclusive results. The first study (Sato et al., 1999) reported a stronger response to scenes than faces between 200 and 300 ms after stimulus onset, with an earlier face-responsive signal observed at ∼170 ms. A more recent study (Rivolta et al., 2012) reported an earlier scene response, peaking between 100 and 130 ms after stimulus onset (M100p), with a face-related component manifesting at the same time window, but at different medial-occipital sites.

Note, however, that no strong conclusions can be made from either of these studies, since they contrasted faces and scenes only, leaving open the possibility that simple visual differences between faces and scenes or a decreased response to faces drive the observed differences rather than a preferential response to scenes. Simply showing a greater response to scenes relative to faces does not specify what type of information is being processed at that time that such a categorical difference manifests. Scenes contain multiple sources of information, ranging from “low-level” image statistics (e.g., spatial frequency and contrast) to “high-level” abstract properties (e.g., scene category or spatial layout; for review, see Groen et al., 2016b), and thus the question of what the actual information is that is being indexed by a putative scene-specific ERP still remains unknown.

Therefore, the present study has the following two goals: (1) to establish the earliest time point at which a preferential response to scenes was observed relative to both faces and objects; and (2) to examine whether the responses at that time point convey scene-specific information. In Experiment 1, participants viewed images of scenes, faces, and everyday objects. We found a positive ERP component peaking at ∼220 ms (P2) after stimulus onset that was stronger for scenes relative to both faces and objects. In Experiment 2, we investigated the sensitivity of the P2 to different types of scene information. We recorded participants’ ERPs while they viewed a rich set of images of naturalistic scenes varying systematically in their global properties (i.e., spatial expanse, naturalness, and relative distance). In a first set of analyses, we found that the P2 amplitude was sensitive to both naturalness and spatial expanse at a categorical level. To explore to what extent the P2 amplitude was modulated by these properties at the level of individual scenes, in a second independent analysis we quantified the naturalness and spatial layout of each scene using both image statistics and behavioral ratings from independent observers. Both the image statistics and behavioral ratings explained significant variance in the P2 amplitude for individual scenes. Importantly, these modulations by individual image characteristics were present only for the P2, and not for the earlier non-scene-selective P1 or N1 components. Together, these results show the emergence of stronger responses to scenes and the presence of global scene information around 220 ms after stimulus onset.

## Materials and Methods

### Subjects

Human subjects were recruited at the Hebrew University of Jerusalem. Twelve students (8 females; age range, 18–28 years) with normal or corrected-to-normal visual acuity and no history of psychiatric or neurological disorders participated in the reported studies. All participants signed an informed written consent form according to the guidelines of the institutional review board of faculty of social sciences of the Hebrew University of Jerusalem and received due compensation for their participation.

### Stimuli

#### Experiment 1: scene selectivity

**Figure 1.**
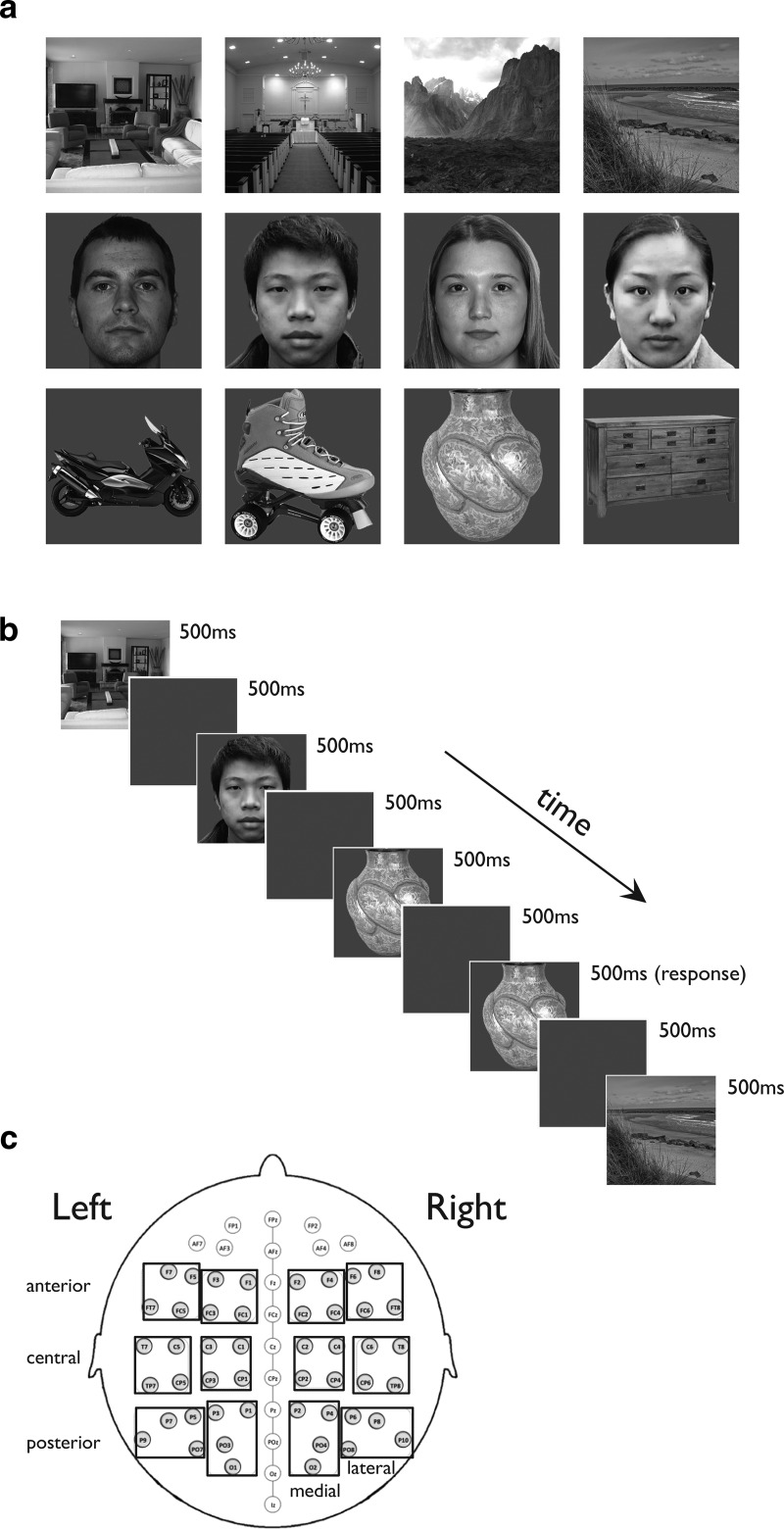
Stimuli and experimental design of Experiment 1 (category selectivity). ***a***, Examples of the stimuli used in Experiment 1. The stimuli consisted of the following three categories: scenes, faces, and objects. Scenes were selected from the following six categories: churches, concert halls, living rooms, beaches, mountains, and deserts (top row, four categories are depicted here). The face stimuli comprised Asian and Caucasian, male and female faces presented in front view (middle row). The objects consisted of dressers, vases, motorbikes, and roller skates (bottom row). Note that in total there were 48 unique exemplars within each visual category. ***b***, Participants viewed the stimuli and performed a simple one-back task, responding whenever the same image was presented twice in a row (in this example, the second presentation of the vase). The stimuli were presented pseudorandomly, with a trial beginning with the presentation of a scene image for 500 ms followed by a blank gray screen for the following 500 ms. ***c***, Schematic representation of the 64 electrode sites from which EEG activity was recorded. The grouped electrodes are those analyzed in the 12 critical regions (see text for details).

The stimuli consisted of 144 grayscale images from the following three visual categories: scenes, faces, and objects (Fig. 1a, stimulus examples). Each visual category contained 48 individual exemplars, spanning multiple subcategories to ensure a wide variety of visual stimulation. The scene stimuli comprised six subcategories, half of them indoor scene categories (churches, concert halls, living rooms), and half of them outdoor scenes (beaches, mountains, deserts). The face stimuli varied in sex and race, comprising Asian and Caucasian, male and female faces presented in front view. The objects consisted of dressers, vases, motorbikes, and roller skates. The mean luminance of all images was equated across categories, with a uniform gray background equated to the mean luminance of the objects. Images were 300 × 300 pixels subtending a square of 8º × 8º at the center of the visual field.

#### Experiment 2: global scene properties

**Figure 2.**
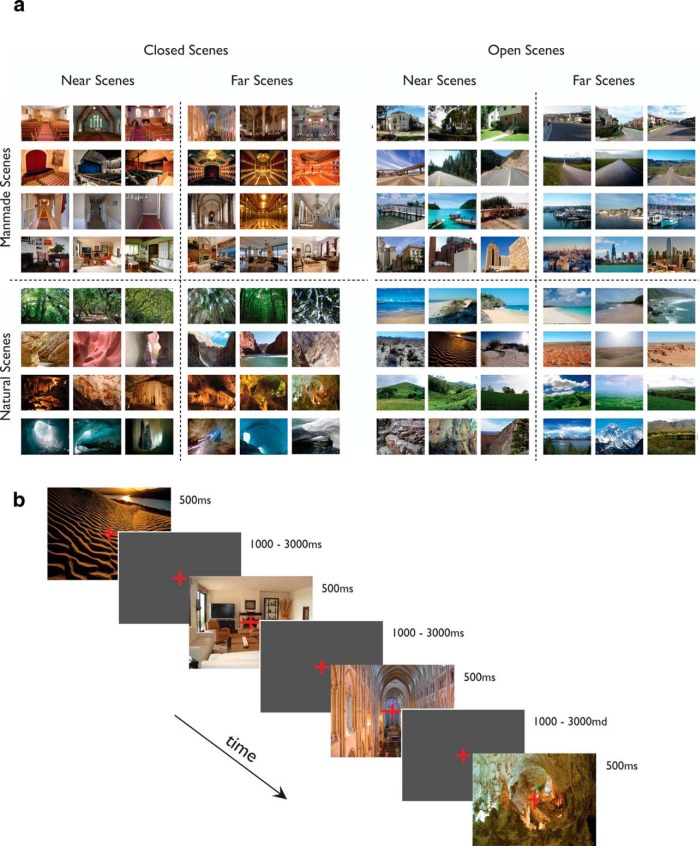
Stimuli and experimental design of Experiment 2 (scene diagnostic properties). ***a***, Full stimulus set. The stimulus set comprised 96 individual, highly detailed, and diverse real-world scene images from 16 basic-level scene categories (churches, concert halls, hallways, living rooms, forest canopies, canyons, caves, ice caves, cities, harbors, highways, suburbs, beaches, deserts, hills, and mountains), with six exemplars within each category spanning the following three diagnostic scene properties: spatial expanse (open, closed; the spatial boundary of the scene); relative distance (near, far; distance to the nearest foreground objects); and naturalness (or semantic content; man-made, natural). ***b***, Participants viewed the stimuli while performing an orthogonal fixation cross task, in which they were required to report whether the horizontal or vertical bar of the central fixation cross lengthened on each trial. Scene stimuli were presented for 500 ms, with a jittered interstimulus interval ranging from 1000 to 3000 ms.

Stimuli for this experiment were images of scenes that had previously been used in a neuroimaging study (Kravitz et al., 2011). The stimulus set comprised 96 individual, highly detailed, and diverse real-world scene images from 16 basic-level scene categories (churches, concert halls, hallways, living rooms, forest canopies, canyons, caves, ice caves, cities, harbors, highways, suburbs, beaches, deserts, hills, mountains), with six exemplars within each category, spanning the following three diagnostic scene properties: spatial expanse (open, closed; the spatial boundary of the scene); relative distance (near, far; distance to the nearest foreground objects); and naturalness (or semantic content; man-made, natural; Fig. 2a, full stimulus set). The images were presented full screen, subtending 27º of visual angle, at a viewing distance of 75 cm. The stimuli were presented using E-Prime presentation software (Psychology Software Tools).

### Experimental design and procedure

Participants were tested in both experiments within the same session. A session consisted of 14 blocks: the first and last blocks of each session were the category selectivity experiment (Experiment 1), and the remaining 12 blocks were the diagnostic scene properties experiment (Experiment 2).

#### Experiment 1

Participants viewed the stimuli and performed a simple one-back memory task (Fig. 1b), responding whenever the same image was presented twice in a row. The stimuli were presented in a pseudorandom order, with a trial beginning with the presentation of an image for 500 ms followed by a gray fixation screen for the following 500 ms. Individual stimuli were presented once within each block.

#### Experiment 2

Participants viewed the 96 scene stimuli, while performing an orthogonal fixation cross task, in which they were required to report whether the horizontal or vertical bar of the central fixation cross lengthened on each trial (Fig. 2b, details). This is the same task used in the prior fMRI study using the same stimuli (Kravitz et al, 2011). The 96 scene stimuli were pseudorandomized within individual blocks and across the 12 blocks. Each scene stimulus was presented once within each block. Scene stimuli were presented for 500 ms with a jittered interstimulus interval ranging from 1000 to 3000 ms.

### EEG recording

The EEG analog signals were recorded for the entire length of the experimental session by 64 Ag-AgCl pin-type active electrodes (ActiveTwo, Biosemi) mounted on an elastic cap (ECI) according to the extended 10-20 system, and from two additional electrodes placed at the right and left mastoids, and an electrode on the tip of the nose. All electrodes were referenced to the common mode signal electrode placed between electrodes PO3 and PO4. Eye movements, as well as blinks, were monitored using two pairs of EOG electrodes, one pair attached to the external canthi, and the other to the infraorbital and supraorbital regions of the right eye. Both EEG and EOG were sampled at 256 Hz with a resolution of 24 bits and an active input range of −262 to +262 mV/bit, with on-line low-pass filtering of 51 Hz to prevent aliasing. The digitized EEG was saved and processed off-line.

### Data processing

The data were preprocessed using the FieldTrip toolbox (Oostenveld et al., 2011). The raw data were first high-pass filtered at 1.0 Hz (24 dB) and referenced to the tip of the nose. Eye movements were corrected using an ocular correction ICA procedure (Jung et al., 1998). Remaining artifacts exceeding ±100 mV in amplitude or containing a change of >100 mV in a period of 50 ms were rejected. The preprocessed data was then segmented into epochs ranging from −250 ms before to 500 ms after stimulus onset for all conditions. Further data analysis was conducted using custom scripts written in Matlab (MathWorks).

### ERP analysis: experiment 1

Twelve separate “regions of interest” were computed from 48 lateral electrodes, each comprising the mean of 4 electrodes (Fig. 1c). These were based on hemisphere, and within each hemisphere they were grouped along a medial–lateral axis and an anterior–posterior axis (Barber et al., 2011). There were six electrode groups in each hemisphere, with two in each of the anterior, central, and posterior scalp sites; one in the lateral position of the hemisphere; and one in the medial position of the hemisphere, as follows: left anterior lateral (F7, F5, FT7, FC5); left anterior medial (F3, F1, FC3, FC1); left central lateral (T7, C5, TP7, CP5); left central medial (C3, C1, CP3, CP1); left posterior lateral (P7, P5, P9, PO7); left posterior medial (P3, P1, PO3, O1); and similarly for the right hemisphere. In the category localizer study, for each subject the peaks of the P1, N1, and P2 for each separate category in each of the electrode groups were determined as the most positive peak between 80 and 130 ms, the most negative peak between 130 and 200 ms, and the most positive peak between 200 and 320 ms, respectively. Differences between mean peak amplitudes (across subjects) were analyzed using a four-way, within-subject ANOVA with hemisphere (left, right), site (anterior, central, posterior), mediality (medial, central), and category (scenes, objects, faces) as independent factors to test the presence of any category-selective effects on the amplitude of the individually defined peaks of each one of the ERP components. For factors with more than two levels, *p* values were corrected for nonsphericity using the Greenhouse–Geisser correction (for simplicity, the uncorrected degrees of freedom are presented; Picton et al., 2000).

### ERP analysis: experiment 2

To avoid a potential bias in our peak selection for Experiment 2, we adopted an ERP “independent localizer” approach, which uses a functional signature from one experiment to determine the latency and loci of the effects of a different experiment (Luck, 2014). Specifically, we defined P1, N1, and P2 time windows for each participant by selecting three time points centered on the peak of the components from Experiment 1 (the time point of the peak, the point prior to it, and the one following it): for P2, the peak was defined as the maximum value between 200 and 320 ms of the difference wave resulting from the subtraction of the object waveform from the scene waveform; for N1, the peak was defined as the maximum value between 130 and 200 ms of the difference wave resulting from the subtraction of the object waveform from the face waveform; and for P1, the peak was defined as the maximum value between 80 and 130 ms of the average waveform formed from averaging the object, scene, and face waveforms. We then extracted the maximal ERP amplitudes in these time windows for each of the conditions of Experiment 2 (the diagnostic scene properties study). Analyses in Experiment 2 were restricted to posterior lateral sites where maximal effects of category were observed. Mean amplitudes were subjected to a four-way ANOVA with hemisphere (right, left), naturalness (man-made, natural), distance (near, far), and spatial expanse (open, closed) as independent variables.

### Single-image EEG analysis

#### Single-image statistics

To investigate the relation between scene properties and image statistics at the level of the individual scenes, we computed two sets of image statistics. The first set of statistics consisted of contrast energy (CE) and spatial coherence (SC). These two parameters are derived from local contrast values (Ghebreab et al., 2009; Scholte et al., 2009) and have previously been shown to predict behavioral performance on man-made versus natural categorization (Groen et al., 2013). In natural scenes, CE and SC typically correlate highly with parameters of a Weibull function fitted to the distribution of contrast values, which reflects the amount of fragmentation in a scene (Simoncelli, 1999; Geusebroek and Smeulders, 2003). CE is a biologically realistic approximation of the distribution width (the scale parameter of the function), whereas SC is an approximation of its shape (the degree to which the function describes a power law or a Gaussian distribution). These two statistics thus capture information about the overall strength of edges in an image (CE) and higher-order correlations between them (SC). Typically, images with high CE values have strong edges due to objects standing out from the background, whereas images with high SC values are cluttered or textured. Here, we computed one CE and one SC value for each scene using the model described previously by Groen et al. (2013).

The second set of statistics consisted of Fourier intercept (FI) and Fourier slope (FS), which are derived from the spatial frequency distributions of individual scenes. These statistics were computed using the procedure described by Oliva and Torralba (2001; i.e., fitting a line to the rotationally averaged power spectrum; but see Groen et al., 2012). These parameters are sensitive to differences in the falloff of the amplitude spectrum of natural scenes and together form a “spectral signature” of an image, which has been shown to be diagnostic of various scene properties (Torralba and Oliva, 2003), including the global scene properties that were manipulated in Experiment 2. To determine whether the global properties were differentially distributed within each of these sets of image statistics, two-sided Kolmogorov–Smirnov tests were conducted for each image parameter (CE, SC, FI, and FS) and global property (naturalness, distance, and spatial expanse) separately.

#### Single-image behavioral ratings

To quantify the degree of naturalness and spatial expanse of individual scenes, behavioral ratings were obtained from a previous experiment using the same set of stimuli (Kravitz et al., 2011). These ratings reflect the level of naturalness or spatial expanse of each scene relative to the others in the set (Kravitz et al., 2011).

#### Single-image ERP analysis

To investigate how the variation in image properties affected the evoked neural activity to individual scenes, we ran hierarchical linear regression analyses of single-image ERP amplitude on the image statistics and behavioral ratings. Per image, single-trial amplitudes at the subject-specific peak time points of the P1, N1, and P2 components identified in Experiment 1 were extracted and subsequently averaged over blocks and subjects, resulting in 96 “single-image” amplitude values per component that were subjected to multilinear regression analysis. In separate analyses, ERP amplitude values were entered as the dependent variable, and the image statistics and behavioral ratings were entered as the independent variables. In the first set of analyses, CE, SC, and naturalness ratings were entered either separately or in combination to predict the ERP amplitude, while in the second set the FI, FS, and spatial expanse ratings were entered. Finally, a third set of analyses was conducted that either included all four image statistics, and naturalness and spatial expanse ratings, or image statistics and behavioral ratings combined. Each regression analysis resulted in a measure of explained variance (*R* ^2^). These analyses were restricted to the electrodes in the posterior lateral sites, as detailed above (which showed maximal scene selectivity) and were performed on ERP amplitude values averaged across the left and right hemisphere.

Table 1 summarizes the statistical analyses conducted in all experiments (Table 1; superscript letters in Results indicate rows in the table). Observed power was calculated *post hoc* with GPower version 3.1 (Faul et al., 2007).

**Table 1:** Summary of key statistical analyses

	Data structure	Type of test	Observed power (α = 0.05)
a	Four-factor, within-subject design: hemisphere, site, mediality, category	Repeated-measures ANOVA	0.99
b	One-way, within-subject design: category	Repeated-measures ANOVA	0.40
c	One-way, within-subject design: category	Repeated-measures ANOVA	0.35
d	Four-factor, within-subject design: hemisphere, site, mediality, category	Repeated-measures ANOVA	0.99
e	Two-factor, within-subject design: category, site	Repeated-measures ANOVA	0.70
f	Two-factor, within-subject design: category, site	Repeated-measures ANOVA	0.40
g	One-way, within-subject design: category	Repeated-measures ANOVA	0.40
h	One-way, within-subject design: category	Repeated-measures ANOVA	0.40
i	Category: faces/objects	Paired *t* test	1.00
j	Category: faces/scenes	Paired *t* test	1.00
k	Category: scenes/objects	Paired *t* test	0.43
l	Two-factor, within-subject design: category, site	Repeated-measures ANOVA	0.70
m	Four-factor, within-subject design: hemisphere, site, mediality, category	Repeated-measures ANOVA	0.99
n	Category: scenes/objects	Paired *t* test	0.99
o	Category: scenes/faces	Paired *t* test	0.99
p	Category: faces/objects	Paired *t* test	0.78
q	Site: posterior/central	Paired *t* test	0.99
r	Site: central/frontal	Paired *t* test	0.99
s	Two-factor, within-subject design: category, component	Repeated-measures ANOVA	1.00
t	One-way, within-subject design: category	Repeated-measures ANOVA	1.00
u	One-way, within-subject design: category	Repeated-measures ANOVA	1.00
v	Category: scenes/faces	Paired *t* test	0.99
w	Category: scenes/objects	Paired *t* test	0.71
x	Category: faces/objects	Paired *t* test	0.61
y	Category: faces/scenes	Paired *t* test	0.96
z	Category: faces/objects	Paired *t* test	0.76
aa	Category: scenes/objects	Paired *t* test	0.12
ab	One-way, within-subject design: category	Repeated-measures ANOVA	1.00
ac	Four-factor, within-subject design: hemisphere, site, mediality, category	Repeated-measures ANOVA	0.99
ad	Spatial expanse (natural scenes): closed/open	Paired *t* test	0.65
ae	Spatial expanse (man-made scenes): closed/open	Paired *t* test	0.12
af	Four-factor, within-subject design: hemisphere, site, mediality, category	Repeated-measures ANOVA	0.99
ag	Four-factor, within-subject design: hemisphere, site, mediality, category	Repeated-measures ANOVA	0.99
ah	One-way, within-subject design (left hemisphere): distance	Repeated-measures ANOVA	0.99
ai	One-way, within-subject design (right hemisphere): distance	Repeated-measures ANOVA	0.90
aj	Two-way, within-subject design (left hemisphere): naturalness, distance	Repeated-measures ANOVA	0.99
ak	Two-way, within-subject design (right hemisphere): naturalness, distance	Repeated-measures ANOVA	0.99
al	4 computational variables (*n* = 96), 2 categorical labels (*n* = 96)	Two-sampled Kolmogorov–Smirnov test (nonparametric)	Not applicable^a^
am	3 response variables (*n* = 96), 6 predictor variables (*n* = 96)	Linear multiple regression (ordinary least squares)	see Table 4^b^

*^a^*95% confidence intervals on actual distribution means are reported in Results.

*^b^Post hoc* power and *R* ^2^ for all regression models are reported in Tables 4 and 5.

## Results

### Experiment 1: category selectivity

Our primary question was whether there is an ERP component that indexes scene-selective processing. We focus here on the first three visually evoked ERP components: P1, N1, and P2 (Key et al., 2005). We first conducted an omnibus four-way, repeated-measures ANOVA on the amplitude of the individually defined peaks of each one of the ERP components with hemisphere (left, right), site (anterior, central, posterior), mediality (lateral, medial), and category (scenes, objects, faces) as independent factors (Table 2, full results of the ANOVA). This data-driven approach includes the vast majority of electrodes (except for mid-sagittal and anterior prefrontal electrodes; Picton et al., 2000) and was adopted given the very limited knowledge of ERP markers of scene selectivity, which precludes an a priori choice of electrode sites or specific components. Nonetheless, based on the reported locus of face selectivity using ERPs (Rossion and Jacques, 2011) and the close proximity of face- and scene-selective areas measured with fMRI, we expected maximal category effects to manifest in the posterior lateral electrode sites. We next describe each component according to their temporal order.

**Table 2:** Experiment 1

Factor	df	MSE (Greenhouse–Geisser)	*F*	Significance
P1 peak amplitudes ANOVA				
Hemisphere	1,11	7.068	0.375	0.553
Category	2,22	5.658	0.504	0.605
Site	2,22	426.203	24.098	0.000
Mediality	1,11	30.689	12.779	0.004
Hemisphere × category	2,22	4.748	3.361	0.061
Hemisphere × site	2,22	3.296	0.265	0.637
Category × site	4,44	9.389	5.164	0.008
Hemisphere × category × site	4,44	0.273	0.301	0.748
Hemisphere × mediality	1,11	0.001	0.001	0.972
Category × mediality	2,22	2.630	3.557	0.054
Hemisphere × category × mediality	2,22	0.518	0.957	0.365
Site × mediality	2,22	8.268	4.748	0.046
Hemisphere × site × mediality	2,22	0.251	0.175	0.741
Category × site × mediality	4,44	0.401	1.384	0.265
Hemisphere × category × site × mediality	4,44	7.068	0.375	0.553
N1/170 peak amplitudes ANOVA				
Hemisphere	1,11	28.032	1.294	0.280
Category	2,22	19.794	1.806	0.189
Site	2,22	27.333	0.797	0.395
Mediality	1,11	4.458	0.674	0.429
Hemisphere × category	2,22	0.645	0.252	0.722
Hemisphere × site	2,22	0.149	0.023	0.942
Category × site	4,44	33.346	3.232	0.079
Hemisphere × category × site	4,44	0.413	0.328	0.746
Hemisphere × mediality	1,11	0.003	0.001	0.976
Category × mediality	2,22	7.202	4.454	0.026
Hemisphere × category × mediality	2,22	0.013	0.024	0.933
Site × mediality	2,22	0.958	0.232	0.669
Hemisphere × site × mediality	2,22	2.160	1.122	0.325
Category × site × mediality	4,44	9.107	7.708	0.002
Hemisphere × category × site × mediality	4,44	0.576	0.941	0.416
P2 peak amplitudes ANOVA				
Hemisphere	1,11	5.056	0.241	0.633
Category	2,22	131.532	10.925	0.001
Site	2,22	533.726	23.344	0.000
Mediality	1,11	17.776	1.309	0.277
Hemisphere × category	2,22	3.147	0.565	0.548
Hemisphere × site	2,22	5.497	1.375	0.271
Category × site	4,44	7.236	2.077	0.146
Hemisphere × category × site	4,44	0.422	0.536	0.650
Hemisphere × mediality	1,11	0.00002	0.000	0.999
Category × mediality	2,22	1.596	0.880	0.418
Hemisphere × category × mediality	2,22	0.741	0.982	0.387
Site × mediality	2,22	0.882	0.463	0.592
Hemisphere × site × mediality	2,22	0.738	0.823	0.434
Category × site × mediality	4,44	0.429	0.660	0.521
Hemisphere × category × site × mediality	4,44	0.548	1.968	0.146

#### P1 component

Overall, analysis of the P1 component^a^ did not show a strong preference for any of the three categories (Table 2, P1 peak amplitudes ANOVA). While category significantly interacted with site [*F*_(2,22)_ = 5.16, mean squared error (MSE) = 1.18, *p* = 0.008], and mediality (*F*_(2,22)_ = 3.55, MSE = 0.74, *p* = 0.05)^a^, *post hoc* analyses of the category effect did not reach significance for either site (anterior: *F*_(2,22)_ = 2.79, MSE = 0.71, *p* = 0.08; central: *F*_(2,22)_ = 1.64, MSE = 1.04, *p* = 0.21; posterior: *F*_(2,22)_ = 0.38, MSE = 1.61, *p* = 0.67)^b^ or mediality (lateral sites: *F*_(2,22)_ = 0.14, MSE = 0.97, *p* = 0.86; medial sites: *F*_(2,22)_ = 1.22, MSE = 1.02, *p* = 0.31)^c^ levels.

#### N1/N170 component

**Figure 3.**
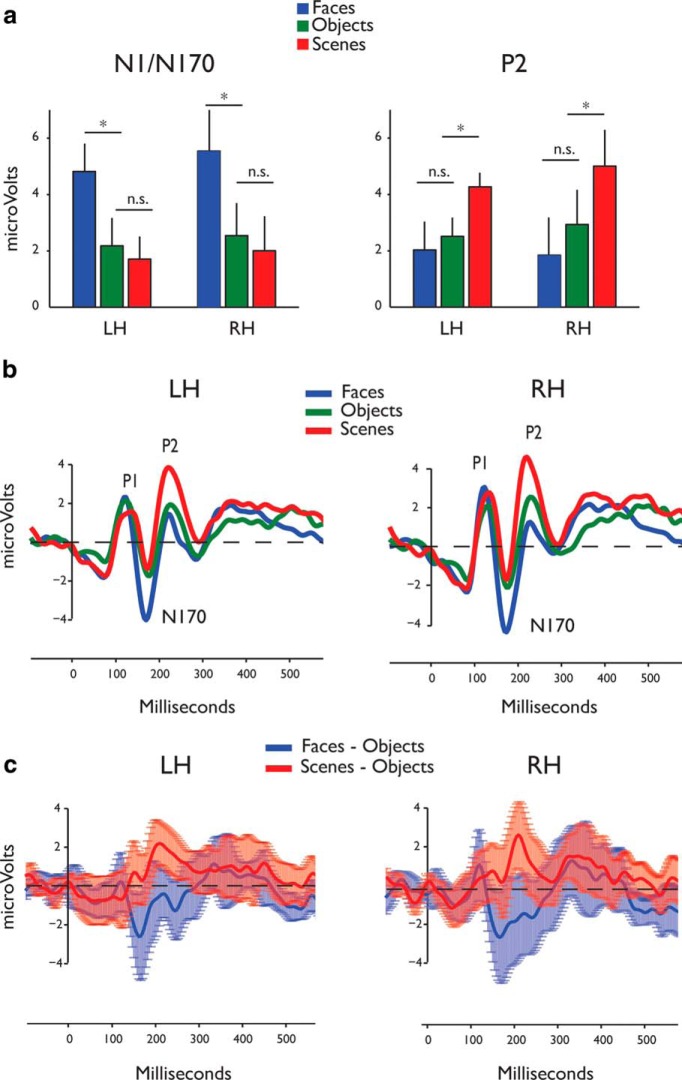
Experiment 1 results. ***a***, Mean N1/170 and P2 peak amplitudes (left and right column, respectively) in response to scenes (red), faces (blue), and objects (green; peak amplitudes are plotted separately for each hemisphere, for the posterior lateral electrode sites. Error bars indicate the SEM. Significant differences (*p* < 0.05) between pairs of categories are denoted by asterisk. ***b***, Group-averaged ERPs (*n* = 12) for the three categories (scenes in red, faces in blue, objects in green) for the left and right hemispheres (data are plotted for the posterior lateral sites). ***c***, ERP difference waveforms depicting face sensitivity (blue, faces-objects) and scene sensitivity (red, scenes-objects) over time for the left and right hemispheres (data are plotted for the posterior lateral sites). The waveforms (solid lines) are presented with across-subjects 95% confidence intervals around them (light blue and red for face and scene sensitivity, respectively).

As expected, the N1 component showed the well known N170 face effect (Bentin et al., 1996), with its strongest amplitude evoked by images of faces relative to images of objects and scenes (Table 2, N1/170 peak amplitudes ANOVA). This effect was most pronounced in posterior lateral electrodes (Fig. 3a, left), as revealed in a significant category × site × mediality interaction (*F*_(4,44)_ = 7.71, MSE = 9.107, *p* = 0.002)^d^. Follow-up category × site ANOVA^e^ for the lateral sites (posterior^f^, central^g^, and anterior^h^) revealed an N170 effect that was restricted to the posterior lateral sites (*F*_(2,22)_ = 7.01, MSE = 4.65, *p* = 0.007)^f^, showing a stronger amplitude to faces (mean = −4.83 mV, SEM = 0.98) than to objects (mean = −2.39 mV, SEM = 0.82; *t*_(11)_ = 2.53, *p* = 0.01)^i^ or scenes (mean = −2.03 mV, SEM = 0.73 *t*_(11)_ = 3.70, *p* = 0.002)^j^, which did not differ in their amplitude (*t*_(11)_ = 0.52, *p* = 0.30^k^; Fig. 3a, left). No significant main effects or interactions were found for the medial sites (Table 2, N1/170 peak amplitudes ANOVA)^l^.

#### P2 component

The first visually evoked component to show a significantly stronger response to scenes compared with objects and faces was the P2 (Fig. 3a, right), peaking at ∼220 ms after stimulus onset (mean = 223 ms, SEM = 3). A main effect of category (Table 2, P2 peak amplitudes ANOVA) was observed (*F*_(2,22)_ = 10.92, MSE = 12.04, *p* = 0.001)^m^, with greater amplitude to scenes (mean = 2.24 mV, SEM = 0.59) relative to objects (mean = 1.37 mV, SEM = 0.60; *t*_(11)_ = 1.94, *p* = 0.03)^n^ and faces (mean = 0.41 mV, SEM = 0.73; *t*_(11)_ = 5.02, *p* = 0.001)^o^, which were also lower in amplitude relative to objects (*t*_(11)_ = 2.70, *p* = 0.005)^p^. A general increase in amplitude was noted going from anterior to posterior sites (main effect of site: (*F*_(2,2)_ = 23.44, MSE = 22.86, *p* = 0.0001)^m^, with posterior sites showing a higher amplitude (mean = 2.98 mV, SEM = 0.81) relative to central sites (mean = 0.90 mV, SEM = 0.62; *t*_(11)_ = 6.07, *p* = 0.0005)^q^, which showed a higher amplitude than the frontal sites (mean = 0.13 mV, SEM = 0.48; *t*_(11)_ = 2.44, *p* = 0.001)^r^.

Finally, to directly pit the differential category sensitivity of the N170 and P2 components, we conducted a two-way ANOVA restricted to the posterior lateral sites with category (faces, scenes, objects) and component (P1, N1, P2) as independent variables^s^ followed by *post hoc* comparisons of the effects^t–aa^. These analyses confirmed the observed “division of labor” in face and scene selectivity between the N170 and P2, respectively (Fig. 3a), with no selectivity observed at the P1 level^ab^ (consistent with a lack of “low-level” physical stimulus effects). Reflecting the formal statistical tests, the effects of stimulus category, particularly scenes, can be clearly observed in the grand average waveforms evoked by scene, face, and object images during the first 500 ms (plotted for the posterior lateral sites; Fig. 3b,c).

The same analyses were performed on the peak latencies of each of the three components reported above, with no category effects or interaction effects found for any of the components.

### Experiment 2: scene diagnostic properties

#### Average ERP analysis

Having established the existence of an early visually evoked ERP component indexing scene selectivity, the P2, with a temporal locus at ∼220 ms after stimulus onset, we next set out to determine the functional properties of the P2 scene-selective component. We asked whether the P2 amplitude captures diagnostic scene information, and if so, what dimensions it is sensitive to. We focused on global ecological scene properties, such as “naturalness” and “spatial expanse,” which have been demonstrated to have psychological reality (Boucart et al., 2013; Groen et al., 2013) as well as neural underpinnings (Kravitz et al., 2011; Park et al., 2011; Harel et al., 2013). Accordingly, we presented naturalistic scene images varying in their spatial expanse, relative distance, and naturalness (Fig. 2), and measured how the amplitude of P2 evoked by these scene images was impacted by changes to each one of these dimensions (for completeness, we also examined the sensitivity of P1 and N1 to these dimensions).

##### P2 component

**Figure 4.**
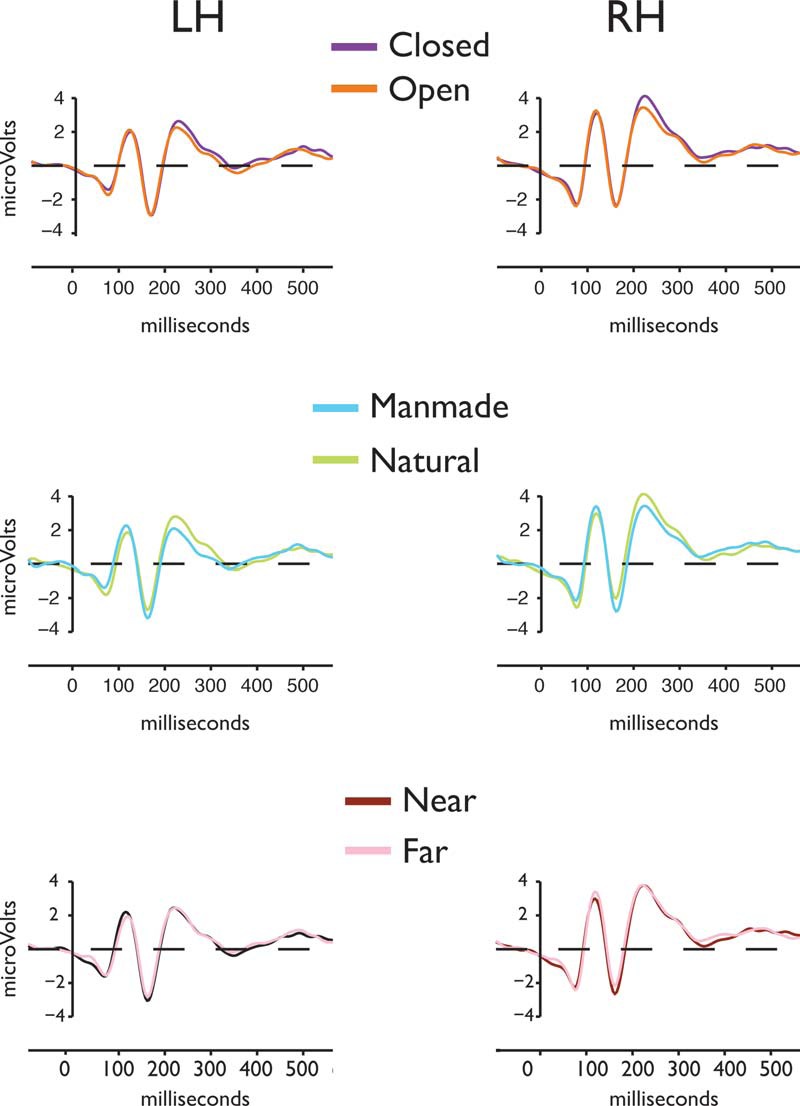
Group-averaged ERPs (*n* = 12) for the three diagnostic scene properties tested in Experiment 2, plotted for the left and right posterior lateral sites. Top row, Spatial expanse (open vs closed). Middle row, Naturalness (man-made vs natural). Bottom row, Distance (near vs far).

**Figure 5.**
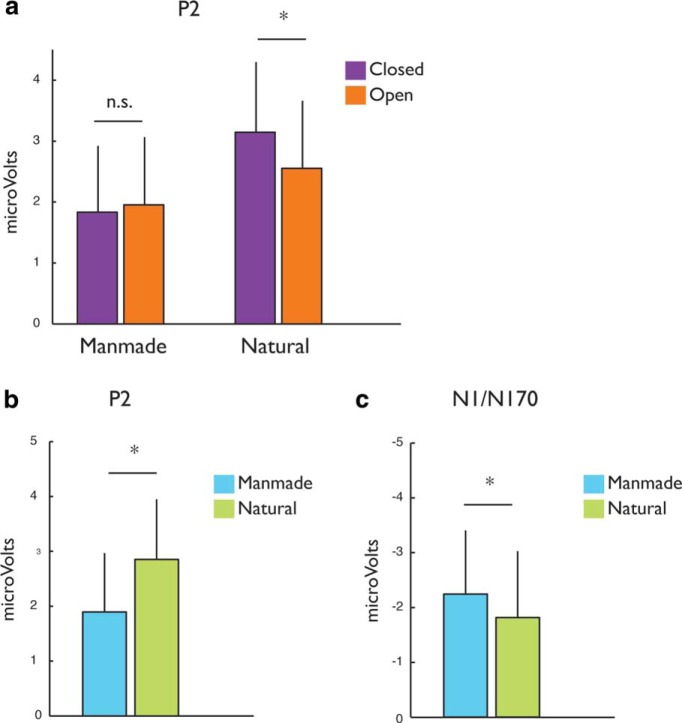
Grand average ERP analysis results for Experiment 2. ***a***, Mean P2 peak amplitudes in response to open and closed scenes (orange and purple, respectively) presented separately for the man-made and natural scenes (left and right columns respectively). ***b***, Mean P2 peak amplitudes in response to natural (green) and man-made scenes (cyan). ***c***, Mean N1 peak amplitudes in response to natural (green) and man-made scenes (cyan). All data are plotted for the posterior lateral sites. Significant differences (*p* < 0.05) between pairs of categories are denoted by asterisk (error bars indicate between-subjects SE).

Using the peak P2 window for each participant identified in Experiment 1 (for details, see Materials and Methods), we extracted amplitudes for each of the eight combinations of the three scene dimensions and submitted them to a four-way repeated-measures ANOVA^ac^ with hemisphere (left, right), naturalness (man-made, natural), distance (near, far), and spatial expanse (closed, open) as independent variables (Fig. 4, grand average waveforms depicting the three main effects; Table 3, P2 peak amplitudes ANOVA, full details of the ANOVA). We observed a significant main effect of naturalness (*F*_(1,11)_ = 26.62, MSE = 1.67, *p* = 0.0005), with natural scenes evoking a greater positive response (mean = 2.85 mV, SEM = 1.05) than man-made scenes (mean = 1.89 mV, SE = 1.02; Fig. 5b). However, this effect was modulated by a significant interaction between spatial expanse and naturalness (*F*_(1,11)_ = 4.59, MSE = 1.32, *p* = 0.05). Follow-up *post hoc* comparisons showed a significant effect of spatial expanse for the natural scenes (*t*_(11)_ = 2.16, *p* = 0.05)^ad^, but not for the man-made scenes (*t*_(11)_ = −0.53, *p* = 0.60)^ae^, with greater positive response for the closed natural scenes relative to the open natural scenes (Fig. 5a).

**Table 3:** Experiment 2

Factor	df	MSE	*F*	Significance
P2 peak amplitudes ANOVA				
Hemisphere	1,11	107.218	2.038	0.181
Naturalness	2,22	43.968	26.223	0.000
Distance	2,22	1.701	1.271	0.284
Spatial expanse	1,11	2.669	1.601	0.232
Hemisphere × naturalness	2,22	0.384	0.554	0.472
Hemisphere × distance	2,22	0.182	0.651	0.437
Naturalness × distance	4,44	0.935	0.438	0.522
Hemisphere × naturalness × distance	4,44	0.686	1.077	0.322
Hemisphere × spatial expanse	1,11	1.422	1.380	0.265
Naturalness × spatial expanse	2,22	6.076	4.593	0.055
Hemisphere × naturalness × spatial expanse	2,22	0.227	0.652	0.436
Distance × spatial expanse	2,22	5.997	2.331	0.155
Hemisphere × distance × spatial expanse	2,22	0.269	0.469	0.508
Naturalness × distance × spatial expanse	4,44	0.308	0.162	0.695
Hemisphere × naturalness × distance × spatial expanse	4,44	0.642	2.025	0.183
N1 peak amplitudes ANOVA				
Hemisphere	1,11	4.177	0.083	0.779
Naturalness	2,22	16.505	4.880	0.049
Distance	2,22	4.211	2.961	0.113
Spatial expanse	1,11	4.006	1.061	0.325
Hemisphere × naturalness	2,22	0.085	0.125	0.730
Hemisphere × distance	2,22	0.066	0.572	0.465
Naturalness × distance	4,44	0.605	0.251	0.626
Hemisphere × naturalness × distance	4,44	0.138	0.922	0.358
Hemisphere × spatial expanse	1,11	1.263	1.204	0.296
Naturalness × spatial expanse	2,22	1.700	0.480	0.503
Hemisphere × naturalness × spatial expanse	2,22	0.197	0.416	0.532
Distance × spatial expanse	2,22	1.283	0.562	0.469
Hemisphere × distance × spatial expanse	2,22	0.042	0.212	0.655
Naturalness × distance × spatial expanse	4,44	6.327	2.176	0.168
Hemisphere × naturalness × distance × spatial expanse	4,44	0.072	0.479	0.503
P1peak amplitudes ANOVA				
Hemisphere	1,11	34.138	0.878	0.369
Naturalness	2,22	6.338	3.015	0.110
Distance	2,22	1.310	0.834	0.381
Spatial expanse	1,11	0.695	0.295	0.598
Hemisphere × naturalness	2,22	0.049	0.058	0.813
Hemisphere × distance	2,22	3.033	10.917	0.007
Naturalness × distance	4,44	4.612	1.135	0.310
Hemisphere × naturalness × distance	4,44	4.332	4.680	0.053
Hemisphere × spatial expanse	1,11	1.265	1.018	0.335
Naturalness × spatial expanse	2,22	2.643	0.437	0.522
Hemisphere × naturalness × spatial expanse	2,22	0.137	0.585	0.460
Distance × spatial expanse	2,22	0.636	0.275	0.610
Hemisphere × distance × spatial expanse	2,22	0.254	0.298	0.596
Naturalness × distance × spatial expanse	4,44	4.428	2.703	0.128
Hemisphere × naturalness × distance × spatial expanse	4,44	0.005	0.010	0.921
P1peak amplitudes ANOVA: *Post hoc* testing of the hemisphere × naturalness × distance interaction				
Left hemisphere				
Naturalness	1,11	0.362	3.641	0.083
Distance	1,11	0.320	0.278	0.608
Naturalness × distance	1,11	0.001	0.001	0.975
Right hemisphere				
Naturalness	1,11	1.106	1.695	0.220
Distance	1,11	0.604	3.449	0.090
Naturalness × distance	1,11	1.391	3.215	0.10

##### N1 component

N1 amplitude also demonstrated a main effect of naturalness (*F*_(1,11)_ = 4.88, MSE = 3.38, *p* = 0.05)^af^. Man-made scenes evoked a greater negative response (mean = −2.20 mV, SE = 1.43) than the natural scenes (mean = −1.61 mV, SE = 1.57; Fig. 5c). No significant interactions were found for any combination of the scene dimensions (Table 3, N1 peak amplitudes ANOVA).

##### P1 component

The P1 component was largely unaffected by the different global scene properties (Table 3, P1 peak amplitudes ANOVA). Interestingly, some interaction effects of distance and hemisphere were noted^ag^. *Post hoc* testing of the hemisphere × distance interaction revealed a significant effect of distance restricted to the left hemisphere (*F*_(1,11)_ = 6.26, MSE = 3.08, *p* = 0.03)^ah^, with a higher amplitude to the near scenes (mean = 3.41 mV, SE = 1.48) relative to the far scenes (mean = 1.62, SE = 0.83). No significant difference was noted in the right hemisphere (*F*_(1,11)_ = 2.76, MSE = 7.71, *p* =0.12)^ai^. *Post hoc* testing of the hemisphere × naturalness × distance interaction^aj, ak^ did not reveal any significant effects in either hemisphere (Table 3, P1 peak amplitudes ANOVA: *post hoc* testing of the hemisphere × naturalness × distance interaction).

#### Single-image ERP analysis

The ERP analysis of the scene stimuli revealed that the spatial expanse and the naturalness of the scene have a direct impact on the P2 scene-selective component. Given the averaging involved in ERP analysis, however, it is difficult to assess using a standard ERP analysis how information contained in individual scene images is processed over time. In order to address this question, we conducted a complementary analysis in which we first quantified the variation in individual scene properties, and then asked whether this variation can explain the variance in the ERP amplitude evoked by individual images.

Specifically, we examined the extent to which the observed differences in peak ERP amplitude are related to differences in image summary statistics, as well as behavioral ratings of the naturalness and spatial expanse of individual images. Summary statistics of scenes can be derived computationally from measurements of spatial frequency and local contrast, and have previously been shown to correlate with several global properties, including spatial expanse and naturalness (Oliva and Torralba, 2001; Kravitz et al., 2011; Groen et al., 2013). We first examined whether these image statistics were correlated with these global distinctions in the particular set of scene images used in Experiment 2. We then tested to what degree the variation in these statistics affected the ERP amplitudes at the single-image level, and to what degree these modulations were shared by behavioral ratings of the images or were uniquely driven by the image properties.

### Image statistics

**Figure 6.**
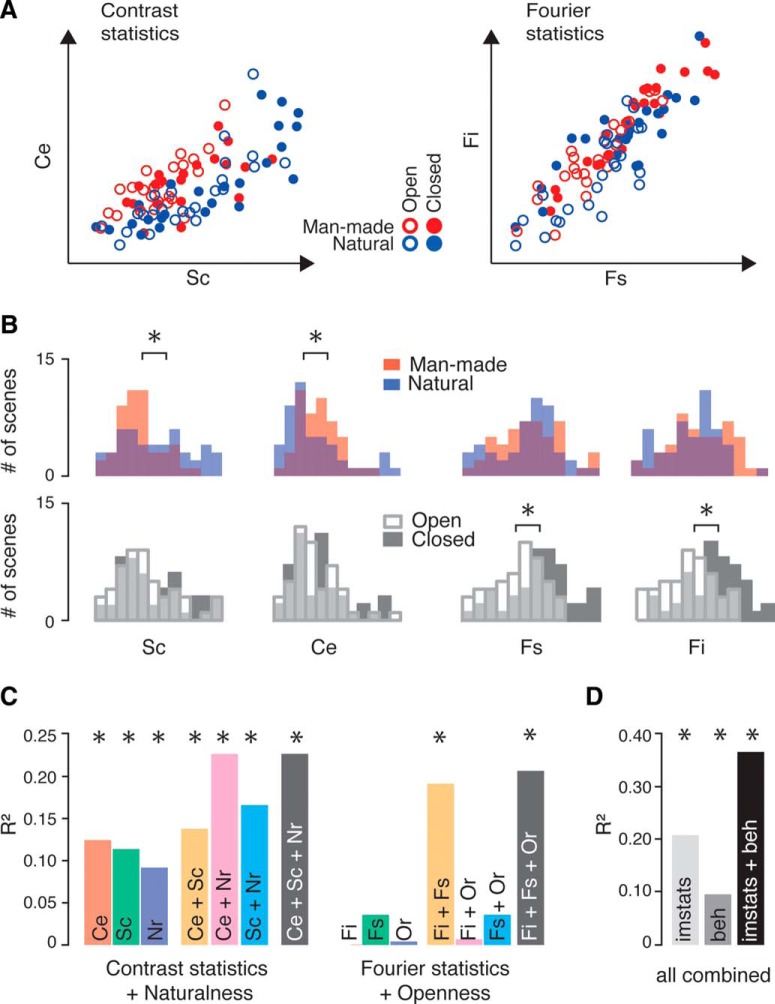
Image statistics analysis of the 96 scene stimuli used in Experiment 2. ***A***, Local contrast statistics (left) CE and SC, and spatial frequency statistics (right) FI and FS (absolute values are plotted for clarity) for each of the 96 scenes, which are color coded by global categorical distinction. The local contrast and spatial frequency statistics describe two-dimensional spaces in which the scenes cluster by naturalness and spatial expanse, respectively. ***B***, The distributions of man-made vs natural, and open vs closed scenes across the four different computational parameters. *p < 0.05. ***C***, Explained variance (*R* ^2^) for a regression model consisting of combinations of local contrast statistics and behavioral naturalness ratings (Nr; left), and a model consisting of spatial frequency statistics and behavioral spatial expanse ratings (Or, right). ***D***, Explained variance for a regression model consisting of all image statistics and all behavioral ratings. Note the change in *y*-axis compared with that in ***C***.

Computational analysis of the 96 scenes based on spatial frequency and local contrast (see Materials and Methods) resulted in two pairs of image statistics parameters describing two “feature spaces” in which images clustered differentially by global property (Fig. 6A). Consistent with previous findings, man-made and natural scenes differed significantly in average contrast energy (Kolmogorov–Smirnov test: *D* = 0.29, *p* = 0.026)^al^ and spatial coherence (*D* = 0.29, *p* = 0.026)^al^, but not in spatial frequency content (FI: *D* = 0.21, *p* = 0.220; FS: *D* = 0.125, *p* = 0.822; Fig. 6B) ^al^. Open and closed scenes, on the other hand, differed in both FI (*D* = 0.44, *p* = 0.0002) ^al^ and FS (*D* = 0.42, *p* = 0.0003) ^al^, but not in contrast energy or spatial coherence (*D* = 0.19, *p* = 0.33; *D* = 0.23, *p* = 0.14, respectively)^al^. No differences in image statistics were found for near versus far scenes (contrast energy: *D* = 0.13, *p* = 0.822; spatial coherence: *D* = 0.15, *p* = 0.65; FI: *D* = 0.19, *p* = 0.333; FS: *D* = 0.13, *p* = 0.822) ^al^. This analysis demonstrates that the global diagnostic scene properties that we found to impact the ERPs at the categorical level can be mapped onto natural image statistics, thereby allowing the quantification of relative variation in this global information between individual scenes.

### Single-image ERP analysis

To examine the extent to which multiple measures of variation in image properties explain modulation of the amplitude of the ERP peaks in which we discerned maximal scene selectivity, we performed a regression analysis of single-image ERP amplitude on the image statistics parameters as well as behavioral ratings of naturalness and spatial expanse obtained previously for these images (see Materials and Methods). We subjected the 96 single-image amplitudes of the scene-selective P2 and the preceding ERP components (P1 and N1) to hierarchical linear regression (see Materials and Methods). Specifically, by comparing the explained variance of models containing both image statistics and behavioral ratings as independent variables (“full models”) with regression models containing separate predictors (“reduced models”), we assessed the unique and shared variance explained by each of these measures. Given the clear relationship we found between naturalness and local contrast measures on the one hand, and between spatial expanse and the Fourier spectrum measures on the other, we first tested the role of each set of summary image statistics relative to its respective behavioral measure of the diagnostic properties, namely the subjective ratings of these properties. Finally, we also examined the joint contribution of image statistics and behavioral ratings by combining all measures.

The results indicated no significant contributions of any regression model (combined or reduced) on the N1 and P1 components (all *R* ^2^ values <0.075, all *p* values > 0.12; Table 4, results of all models). Notably, however, for the P2, significant modulations were found for measures of local contrast statistics, spatial frequency, and behavioral ratings (Fig. 6C). In particular, a model consisting of contrast energy, spatial coherence, and naturalness rating explained 22.7% of the P2 amplitude variance (*F*_(3,92)_ = 8.9, *p* = 0.00003)^am^. This variance was partly shared and partly uniquely explained by these three single-image measures. While each measure was capable of explaining some variance in the P2 amplitude in isolation, combining these measures notably improved the performance of the regression model, with naturalness rating and spatial coherence in particular sharing similar variance. A full model of Fourier intercept, Fourier slope, and spatial expanse ratings also explained a substantial amount of the P2 variance (20.7%, *F*_(3,92)_ = 7.98, *p* = 0.00008)^am^, and, again, a combination of measures was required to achieve the highest performance. In particular, FI and FS were predictive only of P2 amplitude when considered in combination, and spatial expanse rating contributed little additional variance beyond that explained by the spatial frequency measures (Table 5, full results of all regression models for P2). Finally, a full model containing all six measures explained 36.6% of P2 variance, which was more than the image statistics and behavioral ratings explained separately (Fig. 6D).

**Table 4:** Experiment 2 P1 and N1 multilinear regression analysis

Model	df	P1					N1				
*R* ^2^	MSE	*F*	*p*	Power	*R* ^2^	MSE	*F*	*p*	Power
Contrast energy	1,94	0.002	1.56	0.17	0.69	0.07	3.0e-5	2.05	0.0028	0.96	0.05
Spatial coherence	1,94	0.002	1.56	0.20	0.65	0.07	0.0005	2.05	0.05	0.82	0.06
Naturalness rating	1,94	0.019	1.53	1.83	0.17	0.27	0.018	2.01	1.70	0.19	0.25
Contrast energy, spatial coherence	2,93	0.002	1.58	0.11	0.89	0.07	0.002	2.07	0.08	0.93	0.07
Contrast energy, naturalness	2,93	0.021	1.54	0.97	0.38	0.22	0.018	2.04	0.84	0.43	0.19
Spatial coherence, naturalness	2,93	0.028	1.54	1.24	0.29	0.27	0.018	2.04	0.84	0.43	0.19
Contrast energy, spatial coherence, naturalness rating	3,92	0.028	1.55	0.87	0.46	0.24	0.018	2.06	0.56	0.65	0.17
Fourier intercept	1,94	0.009	1.55	0.87	0.35	0.15	0.002	2.05	0.21	0.65	0.07
Fourier slope	1,94	0.017	1.54	1.67	0.19	0.25	0.001	2.05	0.10	0.75	0.06
Openness rating	1,94	0.008	1.54	0.78	0.38	0.15	0.010	2.03	0.99	0.32	0.17
Fourier intercept, fourier slope	2,93	0.019	1.54	0.92	0.40	0.21	0.003	2.07	0.12	0.89	0.08
Fourier intercept, openness rating	2,93	0.012	1.56	0.58	0.56	0.14	0.020	2.03	0.93	0.40	0.21
Fourier slope, openness rating	2,93	0.020	1.54	0.96	0.39	0.22	0.015	2.04	0.71	0.49	0.17
Fourier intercept, Fourier slope, openness rating	3,92	0.024	1.55	0.75	0.52	0.21	0.021	2.05	0.65	0.59	0.19
Contrast energy, spatial coherence, Fourier intercept, Fourier slope	4,91	0.032	1.56	0.75	0.56	0.25	0.005	2.11	0.11	0.98	0.07
Naturalness rating, openness rating	2,93	0.045	1.51	2.17	0.12	0.45	0.021	2.03	0.974	0.38	0.22
All combined	6,89	0.075	1.53	1.21	0.31	0.48	0.036	2.09	0.55	0.77	0.23

**Table 5:** Experiment 2 P2 multilinear regression analysis

	P2					
Model	df	*R* ^2^	MSE	*F*	*p*	Power
Contrast energy	1,94	0.124	2.16	13.35	0.0004	0.95
Spatial coherence	1,94	0.114	2.19	12.06	0.0008	0.93
Naturalness rating	1,94	0.092	2.24	9.49	0.003	0.86
Contrast energy, spatial coherence	2,93	0.138	2.15	7.43	0.001	0.93
Contrast energy, naturalness	2,93	0.226	1.93	13.61	6.5e-6	1.00
Spatial coherence, naturalness	2,93	0.166	2.08	9.25	0.0002	0.97
Contrast energy, spatial coherence, naturalness rating	3,92	0.227	1.95	8.99	2.7e-5	0.99
Fourier intercept	1,94	4.8e-4	2.47	0.045	0.83	0.06
Fourier slope	1,94	0.036	2.38	3.47	0.656	0.46
Openness rating	1,94	0.004	2.46	0.37	0.54	0.09
Fourier intercept, Fourier slope	2,93	0.191	2.02	10.99	5.1e-5	0.99
Fourier intercept, openness rating	2,93	0.007	2.48	0.31	0.73	0.10
Fourier slope, openness rating	2,93	0.036	2.41	1.72	0.19	0.37
Fourier intercept, Fourier slope, openness rating	3,92	0.207	2.00	7.98	8.1e-5	0.99
Contrast energy, spatial coherence, Fourier intercept, Fourier slope	4,91	0.208	2.10	5.99	0.0003	0.99
Naturalness rating, openness rating	2,93	0.096	2.26	4.94	0.009	1.00
All combined	6,89	0.366	1.65	8.58	2.3e-7	1.00

## Discussion

The goal of the current study was to establish the electrophysiological markers of scene recognition by (1) identifying a scene-selective ERP marker and (2) gauging its scene information content. Experiment 1 revealed that the first ERP component to display scene selectivity is the P2 ERP component peaking 220 ms after stimulus onset. Experiment 2 demonstrated that the amplitude of the P2 component could be used to index information about diagnostic global scene properties, supporting the role of P2 as an ERP marker of complex scene processing. Finally, single-image analysis using both computational and behavioral assessment of individual images revealed that the P2 components, but not the P1 and N1, are sensitive to variation in global properties between scenes.

We started this investigation by searching for the “scene analog” of the face-selective N170 ERP component. We adopted an fMRI-style approach, which incorporated a “functional localizer” (Saxe et al., 2006) to identify a scene-selective ERP component (Experiment 1), followed by an independent experiment to test the functional properties of this component (Experiment 2). Importantly, the functional localizer approach also has the advantage of avoiding the problem of the use of the same dataset for selection and selective analysis known as “double dipping” in the fMRI literature (Kriegeskorte et al., 2009) and “multiple implicit comparisons” in the ERP literature (Luck, 2014, p. 328). Admittedly, the downside of this approach is that it might miss out on more subtle types of effects, which might manifest “outside” of the focus of interest; that is, at different time windows or electrode sites than the ones examined in the independent experiment. The reason to search for such a scene analog comes from the anatomical proximity of the representations of faces and scenes. In humans, a scene-selective region known as the parahippocampal place area (PPA; Epstein and Kanwisher, 1998) can be found on the ventral surface of occipitotemporal cortex close to the face-selective fusiform face area& (Kanwisher et al., 1997), and on the lateral surface of occipitotemporal cortex a scene-selective occipital place area (Dilks et al., 2013) can be found adjacent to a face-selective occipital face area (Gauthier et al., 2000). A similar spatial arrangement of face- and scene-selective regions has also been reported in the monkey (Nasr et al., 2011; Kornblith et al., 2013). This organization has been suggested to reflect distinctive yet complementary computations, such as visual field biases (Levy et al., 2001; Silson et al., 2015; Verhoef et al., 2015), stimulus rectilinearity (Nasr et al., 2014), and spatial frequency content (Rajimehr et al., 2011). Here we observed that this close relationship between the representations of faces and scenes can also be observed temporally, with close temporal proximity in the visual processing for faces (N170) and scenes (P2).

Very little is known about the P2 component (Luck, 2014). Prior literature focused on the processes underlying the P2 in selective attention, primarily in modulation of nontarget stimuli (for review, see Key et al., 2005). Importantly, the current P2 scene-selective effect is not likely to index attentional processes, as all categories appeared with equal probability, and the orthogonal task we used minimized the possibility that attention was differentially allocated across categories. At the same time, we did not find any category effects on the P1 component, which likely indexes early sensory processing within the extrastriate cortex (Clark et al., 1994). This lack of a P1 effect arguably reflects the wide range of stimuli we used in Experiment 1 (for details, see Materials and Methods), selected so as to minimize the possibility that a single stimulus parameter could drive any potential category effects. The lack of earlier scene selectivity suggests that the selectivity in the P2 cannot be reduced to very basic local visual features (e.g., differences in the retinotopic extent of the full-field scenes relative to isolated objects), although such a contribution cannot be ruled out (for a similar logic applied to the N170, see Bentin et al., 2007). Further support for the idea that scene selectivity manifests at the time window of the P2 comes from two recent intracranial studies (Bastin et al., 2013a,b). These studies reported a scene-selective increase in gamma-band activity between 200 and 500 ms after stimulus onset in posterior parahippocampal electrodes, consistent with our findings, as well as with the scene-selectivity of PPA.

To uncover the underlying processes indexed by the P2, in Experiment 2 we examined how its amplitude is modulated by variations in global scene properties that are known to be diagnostic for scene categorization (Greene and Oliva, 2009a). First, a classic grand average ERP analysis revealed that the P2 amplitude is sensitive to whether the scene is natural or man-made, an effect that was further modified by the spatial expanse of the scene. Second, a single-image ERP analysis established that diagnostic scene information has an impact on the P2 not only at the category level, but also at the individual scene image level. Despite the overt differences between these two analyses, the results of the single-image analysis are consistent with the average-based ERP analysis in pointing to the P2 as the critical time window for the integration of information about multiple scene dimensions. We found that two types of natural image statistics (derived from local contrast and the image power spectrum, respectively) could predict the P2 amplitude elicited by single-scene images, and that the variance explained by these factors was partly shared with behavioral ratings of naturalness and spatial expanse of the scenes. Thus, these analyses converge to emphasize the significance of the P2 time window—at ∼220 ms after stimulus onset—for processing of scene-diagnostic information. Notably, converging with our current findings, a recent MEG study reported a marker of real-world scene size ∼250 ms after stimulus onset (Cichy et al., 2016).

The fact that P2 amplitude is sensitive to both scene naturalness and spatial expanse establishes its potential utility for understanding the usage of diagnostic information for scene recognition, as both are considered key global properties for scene categorization (Torralba et al., 2006). Naturalness (or semantic content), in particular, has been suggested to play a central role in scene categorization (Oliva and Torralba, 2001; Greene and Oliva, 2009a; Groen et al., 2013; Berman et al., 2014). For example, the discrimination between man-made and natural scenes occurs rapidly, and precedes categorization based on the basic-level category of the scene (Joubert et al., 2007; Loschky and Larson, 2010; Kadar and Ben-Shahar, 2012; Banno and Saiki, 2015). Indeed, in the current study naturalness already had an effect on the N1 component, albeit only at the grand average analysis level. The N1 time window (150-180 ms) has been highlighted by several works that have looked at categorization of natural scene images using behavioral and electrophysiological measures (for review, see Fabre-Thorpe, 2011). Specifically, an anterior ERP waveform carried information about natural and man-made objects and scenes as early as 150 ms after stimulus onset (Thorpe et al., 1996). This raises the possibility that scene-specific processing, particularly as it relates to the distinction between natural and man-made scenes, can be observed earlier than the P2 level. Note, however, that these studies are typically focused on the recognition of a specific object in a scene, rather than on processing the global diagnostic properties of scenes, as was the focus of the current study. It should also be noted that in contrast to the grand average level analysis, at the single-image level analysis, none of the global statistics we tested were found to have a significant effect on the N1 amplitude. Earlier studies examining the effects of global scene statistics along the entire ERP time course (on a time point-by-time point basis; Groen et al., 2016a) reported that across the whole scalp, naturalness could be decoded as early as 100 ms after stimulus onset. However, consistent with the present results those studies showed that man-made/natural differences only appeared at 200 ms at the posterior lateral sites in which scene selectivity was observed here.

Future work on the significance of P2 as a marker of scene recognition processes will enable to determine how higher-order image properties are combined to form the basis for scene categorization, and how this information varies relative to other visual categories. Given their direct link with the P2 amplitude, the summary image statistics we used here have the potential to provide valuable insights regarding the time course of visual categorization. Specifically, it seems reasonable to assume that the low-level properties that these summary image statistics are derived from (contrast, spatial frequency) are common to all stimulus types (faces, objects, scenes). However, it may be argued that the variation in these image properties is unique to scenes, as these second-order statistics are diagnostic only for scene-related categorization tasks. Put differently, faces and objects might be considered as very particular types of “scenes,” with a very narrow range of contrast and spatial frequency distributions, whereas real-world scenes, in contrast, vary over a much larger range of this information. It is exactly these variations in statistical properties that might be picked up by specialized scene-selective mechanisms. Along these lines, PPA has been reported to respond preferentially not only to real-world scenes, but also to surface textures and object ensembles (Cant and Goodale, 2007; Cant and Xu, 2012). What is common to these seemingly distinct types of stimuli? It has been suggested that the processing of all three types of stimuli requires the extraction of summary statistics without encoding each repeating element in great detail (Cant and Xu, 2012). Thus, some aspects of scene perception may be achieved by a general mechanism for extracting summary image statistics from multiple sources, computing the variation across the entire visual field without necessarily encoding the detailed local features (Oliva and Torralba, 2001; Torralba and Oliva, 2003; Wolfe et al., 2011). At this point, the current conjecture is still speculative and requires more research before establishing strong conclusions. However, one fruitful direction might be the generation of artificial stimuli varying in their summary image statistics (Groen et al., 2012) and assessing how they impact the P2 magnitude.

### Summary

In two experiments we have identified the posterior visual P2 ERP component as the earliest marker of scene selectivity, peaking 220 ms after stimulus onset. The scene-selective P2 effect reflects the processing of diagnostic scene information as we found it to be modulated by two global scene properties: spatial expanse and naturalness. Further, image statistics diagnostic of the global scene properties and behavioral ratings of individual images were predictive of the P2 response. Together, these results suggest that higher-order scene properties become maximally represented ∼220 ms after stimulus onset, and establish the P2 as an ERP marker for scene processing.

## Synthesis

The decision was a result of the Reviewing Editor Lila Davachi and the peer reviewers coming together and discussing their recommendations until a consensus was reached. A fact-based synthesis statement explaining their decision and outlining what is needed to prepare a revision is listed below. The following reviewer(s) agreed to reveal their identity: H.Steven Scholte

This is a very complete set of studies on the temporal dynamics of scene processing, filling a gap in the literature regarding scene processing. The set of two experiments are technically sounded, and the results strong: spatial layout properties modulating the P2 (∼ 220 msec) is a very neat novel result for the field of cognitive neuroscience.

I did read the entire paper, and I must say: this same paper could be cut by 40-50%, with lots of statistics (like comparison with image statistics models/algorithms) that could go in supplement. the paper is lengthy and difficult to read, because it does repeat a lot the same conclusions, findings, etc. My main revision would be to reduce the paper greatly, so readers can enjoy it more. The message is direct and straightforward: a P2 spatial scene component ∼ 220 msec.

The authors first isolate moments in time / locations at which scenes differ from objects and faces. Next they use there moments of interest to explore a second dataset which contains images on multiple axis relevant for scene recognition.

The authors show that the P2 coincides with a moment at which both a different in open / closed and manmade / natural can be found. From this they conclude that the P2 relevant for scene recognition and might be a homologue to the face N170. The paper presented an interesting and nice read. I think the authors are selling themselves short with the actual presentations.

Major comments

•The authors could provide a somewhat gentler introduction to the problem of scene statistics vs. scene recognition. In the end the reasoning in the discussion, that a true scene recognition component should contain sensitivity to multiple scene statistics elements could be made more explicit in the introduction. E.g. the authors should establish in the introduction what a litmus test is for 'true' scene specific information. Now they ignore the problem.

•The moments in time approach gives a clear focus, however, some elements of the data get blurred. For instance at page 17 the authors state there are no low-level physical stimulus effects at the P1. This is of course correct but they could be (and probably are) present in the data as a whole. The authors could provide somewhat more meta info about the general (strong) approach but also note the weakness in the discussion (e.g. what is not found in exp1 is not reported in exp 2).

•The correlations between Ce and Sc and Fi and Fs appear to be very high. But this can be related to the specific image set. Is it? How are the correlations between Ce/Cs vs. Fi/Fs?

•The authors observe that Fi/Fs is related to open / close (following Kravitz) and Ce/Cs related to manmage / natural (following Groen). They find correlations of both in the P2. It should be possible to make the point of this relationship more directly. Can these values be used, as mediation parameters, to reduce the manmade / natural and open / close effects reported in fig 5?

•What is the combined explained variance of Fi+ Fs and Ce + Sc? If both play a different role the combined value should be higher.

Minor comments

•I miss to what degree the authors use mcc for exp 1. This is not necessary if it only pertains to ROI selection but the authors also report the data from exp 1 as a finding by itself. Please clarify.

•P8. For inspection during recording you want to select an actual reference to get the full CMRR (not relevant for recording in the past but might be sensible for the future).

•Include the nose reference (if used) as a measured electrode in EEG recording.

•Bottom p29, one of the Groen papers shows sensitivity to manmande / natural during the N1 amplitude (at least in terms of time) and for SC or CE.

•Some of the references are not in the literature list
